# Evaluation of abdominal compression–decompression combined with chest compression CPR performed by a new device: Is the prognosis improved after this combination CPR technique?

**DOI:** 10.1186/s13049-022-01036-y

**Published:** 2022-08-13

**Authors:** Haishan Li, Chao Wang, Hongyuan Zhang, Fang Cheng, Shuang Zuo, Liyou Xu, Hui Chen, Xiaodong Wang

**Affiliations:** 1 Department of Emergency, The Second People’s Hospital of Hefei, The Affiliated Hefei Hospital of Anhui Medical University, Hefei, China; 2 Center of 120 Emergency, Hefei, China; 3 Department of Nursing, The Second People’s Hospital of Hefei, Hefei, China; 4 Department of Emergency Intensive Care Unit, The Second People’s Hospital of Hefei, Hefei, China

**Keywords:** Out-of-hospital cardiac arrest, Resuscitation, Chest compression, Abdominal compression–decompression, Prognosis

## Abstract

**Introduction:**

This study was designed to compare the outcomes of standard cardiopulmonary resuscitation (STD-CPR) and combined chest compression and abdominal compression–decompression cardiopulmonary resuscitation (CO-CPR) with a new device following out-of-hospital cardiac arrest (OHCA). Moreover, we investigated whether patient prognosis improved with this combination treatment.

**Methods:**

This trial was a single-centre, prospective, randomized trial, and a blinded assessment of the outcomes was performed. A total of 297 consecutive patients with OHCA were initially screened, and 278 were randomized to the STD-CPR group (n = 135) or the CO-CPR group (n = 143). We compared the proportions of patients who achieved a return of spontaneous circulation (ROSC), survived to hospital admission and survived to hospital discharge. In addition, we also performed the Kaplan–Meier analysis with a log-rank test at the end of the follow-up period to compare the survival curves of the two groups.

**Results:**

The differences were not statistically significant in the proportion of patients who achieved ROSC [31/135 (23.0%) versus 35/143 (24.5%)] and survived to hospital admission [28/135 (20.7%) versus 33/143 (23.1%)] between the CO-CPR group and STD-CPR group. However, there was a significant difference in the proportion of patients who survived to hospital discharge [16/135 (11.9%) versus 7/143 (4.9%)] between the two groups. Nine patients (6.7%) in the CO-CPR group and 2 patients (1.4%) in the STD group showed good neurological outcomes according to the cerebral performance category (CPC) scale score, and the difference was statistically significant (*P* = 0.003). The Kaplan–Meier curves showed that the patients in the CO-CPR group achieved better survival benefits than those in the STD-CPR group at the end of the follow-up period (log-rank *P* = 0.007).

**Conclusion:**

CO-CPR was more beneficial than STD-CPR in terms of survival benefits in patients who have suffered out-of-hospital cardiac arrest.

*Trial registration* Chinese Clinical Trial Registry, registered number: ChiCTR2100049581. Registered 30 July 2021- Retrospectively registered. http://www.medresman.org.cn/uc/index.aspx.

**Supplementary Information:**

The online version contains supplementary material available at 10.1186/s13049-022-01036-y.

## Background

Cardiac arrest is a severe, life-threatening condition and remains a leading cause of out-of-hospital death worldwide. Adult patients who experience out-of-hospital cardiac arrest (OHCA) have a low survival rate, approximately 10.4%, and only 8.2% of them survive and have a good functional status [1]. Standard cardiopulmonary resuscitation (STD-CPR), consisting of chest compression and artificial ventilation, is considered the standard treatment for OHCA [1–3]. Conventional chest compression does not always lead to a perfusion pressure that is sufficient to maintain vital organ blood flow and does not always fully restore cardiac and brain function [4–7]. For years, clinicians have pondered how to increase or maintain vital organ blood flow during CPR.

Abdominal compression–decompression cardiopulmonary resuscitation could augment blood return and cardiac output by increasing or reducing the patient’s abdominal pressure. This method not only achieves the conventional effect but can also be used for patients with chest compression contraindications such as chest wall deformity, rib fracture, or haemopneumothorax. Several studies have shown that the abdominal compression–decompression technique is associated with increased coronary perfusion pressure and cerebral blood flow, which can lead to improved survival [8–11]. However, this technique has been evaluated only with animal studies and case reports. In addition, this method is mainly performed manually and lacks any objective and visual parameters, such as the force or depth of the compression–decompression; it can also be difficult to accurately perform this technique. An abdominal CPR compression–decompression instrument, as an external and easy-to-use device, has the potential to be useful in out-of-hospital emergency medical services [11, 12]. The aim of this randomized controlled trial was to determine whether the combined use of conventional chest compression and abdominal compression–decompression techniques improved outcomes in out-of-hospital cardiac arrest patients.

## Materials and methods

### Study design

This study was conducted at the Hefei Second People's Hospital, Hefei, China, between 1 January 2020, and 31 December 2020. Hefei Second People's Hospital, a national CPR training centre in China, is a large referral hospital and a tertiary A-level hospital that has 2583 inpatient beds and over 2 million annual emergency and outpatient visits. Our hospital health service manages out-of-hospital health emergencies in Hefei city and rescues more than 350 patients who experience OCHA each year. Our CPR team consists of two systems. The first system, which performed STD-CPR or CO-CPR out of the hospital, consists of six emergency rescue stations. Each team in the station consists of two junior emergency physicians, one paramedic, and one ambulance driver. The second system, which performed advanced life support, consists of one senior emergency physician, six junior emergency physicians, a head nurse, and several registered nurses from the emergency intensive care unit (EICU). All members were trained to perform two CPR methods according to the American Heart Association guidelines. Abdominal compression–decompression training was performed under the supervision of the manufacturer’s monitoring staff.

The trial was retrospectively registered in the Chinese Clinical Trial Registry (registered number: ChiCTR2100049581). The study was approved by the Ethics Committee of the Second People’s Hospital of Hefei (The Affiliated Hefei Hospital of Anhui Medical University, approval number 2020-Science-025), and the requirement for informed consent was waived. This trial is a single-centre, prospective, randomized trial in which chest compression CPR after OHCA will be compared with a method that combines chest compression and abdominal compression–decompression CPR.

### Patients

The inclusion criterion was OHCA in patients at least 18 years of age. The exclusion criteria were patients aged 80 years or older or patients with any contraindications for the abdominal compression–decompression technique, including pregnancy, history of recent thoracic or abdominal trauma/surgery, known terminal or end-stage disease, or severe neurologic impairment.

### Randomization and blinding

Randomization was performed by a statistics professional from our hospital. A random number table was generated using SPSS 18.0 software. Numbers from the table were assigned on a unified basis by the professional. A blinded assessor evaluated the patient’s neurological prognosis. The outcome assessors and trial statisticians were also blinded. The clinical team responsible for the participants (physicians, nurses, and others) and involved with direct patient care were not blinded to the allocation group due to the inherent difficulty in blinding the intervention.

### Data collection and follow-up

Data were recorded following Utstein resuscitation registry templates, including baseline characteristics, witnesses, bystander CPR, first monitored rhythm, epinephrine use, aetiology, event location and comorbidities.

All patients who were included in this study had a primary cardiac arrest, but cardiac arrests that occur outside the hospital are not always witnessed immediately. Therefore, the exact time from cardiac arrest to the initiation of CPR may not have been accurately determined in all patients in our study; therefore, the no-flow time was waived. Patients were followed-up by three methods: outpatient visits, inpatient visits, or telephone calls. The patients were followed up by a blinded assessor every 1 to 3 months after discharge from the hospital, and the endpoint of the follow-up was the date of death or July 30, 2021.

### Outcome measures

The primary outcome measure was the return of spontaneous circulation (ROSC). Secondary outcome measures were survival to hospital admission, survival to hospital discharge, and neurological outcomes at hospital discharge.

Neurological outcomes were assessed by the Cerebral Performance Category (CPC) scale. The CPC scale categorizes neurological outcomes as follows: CPC 1, good performance; CPC 2, moderate disability; CPC 3, severe disability; CPC 4, comatose or persistent vegetative status; and CPC 5, brain death or patient death [13]. The prognosis in the two groups was compared by classifying the CPC 1 or 2 patients as having a good neurological outcome and those patients with a CPC ≥ 3 as having a poor neurological outcome.

### Sample size

We referred to another similar study at the time of the sample size estimation for this study [12]. The study had 80% power to find a significant result with a threshold two-sided p value of 0.05 if the expected proportion of ROSC was approximately 20%. The sample size was re-estimated as 122 patients for each group, and the final sample size was increased to approximately 150 patients due to a dropout rate of 20% (for details, see the supplementary materials).

### Instrument and intervention

#### Abdominal CPR compression–decompression instrument

The instrument, produced by Beijing Germari Medical Equipment Co., Ltd., consisted of three components: a display panel, pressure application handles, and a negative pressure device. A compression plate on the bottom of the negative pressure device had to be placed on the epigastrium. After turning on the device, negative pressure was generated, which caused a tight bond between these compression plates and the patient's abdomen. When we performed compression, the compression force was approximately 186 mmHg when the indicator light was on. While decompression was performed, the decompression force was approximately 112 mmHg.

**Fig. 1:**
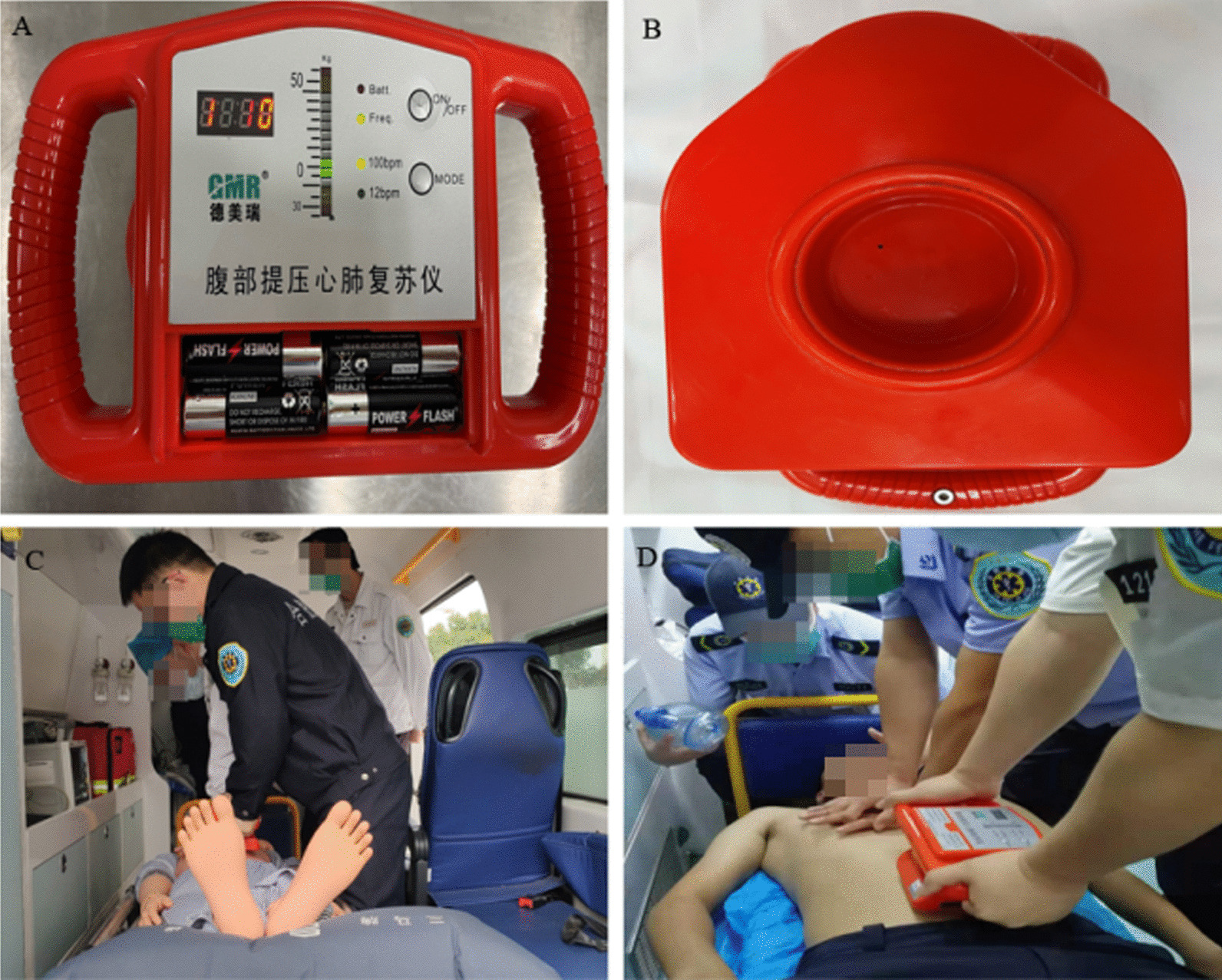
The abdominal compression–decompression device and its use. **A**, **B** The abdominal compression–decompression device. **C** The training of emergency physicians. **D** The performance of CO-CPR

The operating parameters were as follows: (1) The abdominal compression force was limited to 50 kg, and the decompression force was limited to 30 kg; the force levels were controlled from a light-emitting diode (LED) display panel on top of the instrument. (2) The compression–decompression frequency was marked by an audio signal with a frequency of 100 times/min. Images of the device are shown in Fig. 1.

#### Interventions

In principle, CPR attempts were performed according to current American Heart Association guidelines [1]. Patients were randomized to receive either CO-CPR or STD-CPR treatment. For CO-CPR, chest compression was performed in alternation with abdominal compression–decompression, i.e., when the chest was compressed, the abdomen simultaneously would be decompressed, and vice versa. Abdominal compression was performed at a rate of 100 times/min and a depth of 5–10 cm. The rate of abdominal compression–decompression cycles to chest compression cycles, marked by the audio signal and delivered in alternation, was set to 1:1. The effectiveness, safety, and stability of the abdominal compression–decompression device used in this study have been verified in human studies [11, 12].

All methods were performed following relevant regulations and guidelines. Defibrillation was administered as needed. All patients were ventilated with a bag-valve mask during resuscitation during the out-of-hospital period. After referral to the hospital, all patients received orotracheal intubation and respiration with the aid of a rebreathing bag. Targeted temperature management with a target temperature of 33 °C was performed in each patient. The two CPR methods were applied until either ROSC or resuscitation was deemed futile by the treating emergency physicians.

### Statistical analysis

PASS 15 software was used to calculate the sample size (NCSS, LLC., Kaysville, Utah, USA). Statistical analysis was performed using SPSS version 18.0 (SPSS Inc., Chicago, IL, USA) and GraphPad Prism 8.0 (GraphPad Software, La Jolla, California, USA). The continuous variables are presented as the mean ± standard deviation (SD) and were analysed by the independent samples t test, while primary and secondary outcome analyses were conducted using Fisher’s exact or Pearson chi-square (${x}^{2}$) tests for comparison. Kaplan–Meier analysis with the log-rank test was plotted to compare the survival curves of the two groups at the end of the follow-up. A p value less than 0.05 was considered statistically significant.

## Results

### Patients

**Fig. 2:**
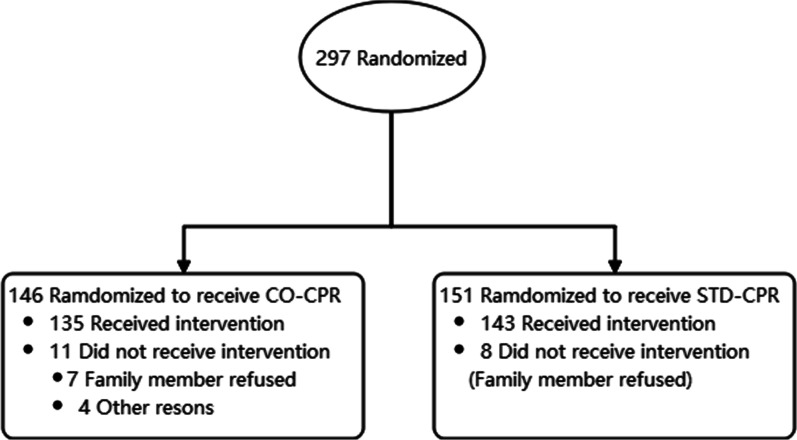
Flowchart for patient selection

A total of 297 consecutive patients with OHCA were initially screened, and 278 were randomized to the STD-CPR group (n = 135) or the CO-CPR group (n = 143). The patient selection and reasons for exclusion are shown in Fig. 2. The patients’ baseline characteristics were similar in the two groups (Table 1).

**Table 1:** Demographic characteristics of the STD-CPR and CO-CPR groups

	CO-CPR	STD-CPR	*P* value*
	Total (n = 135)	Total (n = 143)	
Baseline characteristics			
Age(Mean ± SD), years	58.33 ± 14.88	59.62 ± 14.91	0.471
Men, n(%)	100(74.1%)	101(70.6%)	0.521
Witnessed, n(%)	81(60.0%)	84(58.7%)	0.831
Bystander CPR, n (%)	51(37.8%)	50(35.0%)	0.626
First monitored shockable rhythm, n (%)	11(8.1%)	17(11.9%)	0.300
Epinephrine < 5 mg, n(%)^a^	30(22.2%)	39(27.3%)	0.330
Etiology			
Medical, n (%)	110(81.5%)	108(75.5%)	0.228
Asphyxia, n (%)	14(10.4%)	12(8.4%)	0.571
Drowning, n (%)	0	2(1.4%)	0.499
Others, n (%)	11(8.1%)	21(14.7%)	0.088
Location			
Home, n (%)	85(63.0%)	99(69.2%)	0.270
Public place, n (%)	23(17.0%)	18(12.6%)	0.296
Others, n (%)	27(20.0%)	26(18.2%)	0.700
Comorbidities			
Diabetes, n (%)	48(35.6%)	50(35.0%)	0.918
Hypertension, n (%)	64(47.4%)	63(44.1%)	0.575
Malignancy, n (%)	14(10.4%)	13(9.1%)	0.719
Lung disease, n (%)	7(5.2%)	10(7.0%)	0.530
Previous stroke, n (%)	17(12.6%)	25(17.5%)	0.255
Chronic renal disease, n (%)	8(5.9%)	12(8.4%)	0.427
Cardiovascular disease, n (%)	77(57.0%)	69(48.3%)	0.143
COPD, n(%)	3(2.2%)	4(2.8%)	0.760

COPD, Chronic obstructive pulmonary disease

*Fisher’s exact or Pearson’s chi-square tests

^a^Epinephrine dose dichotomized according to median

### Primary outcome

In the primary analysis, 31 (23.0%) patients in the CO-CPR group and 35 (24.5%) patients in the STD-CPR group achieved ROSC (*P* = 0.767).

### Secondary outcomes and follow-up

We also compared the survival to hospital admission proportions, survival to hospital discharge proportions, and neurological outcomes at hospital discharge in the two groups. Twenty-eight patients (20.7%) in the CO-CPR group and 33 patients (23.0%) in the STD-CPR group survived to hospital admission (*P* = 0.638). However, there was a significant difference in the survival to hospital discharge proportions [16/135 (11.9%) vs. 7/143 (4.9%)] between the two groups. Nine patients (6.7%) in the CO-CPR group and 2 patients (1.4%) in the STD-CPR group achieved good neurological outcomes according to their CPC status (*P* = 0.003).

**Fig. 3:**
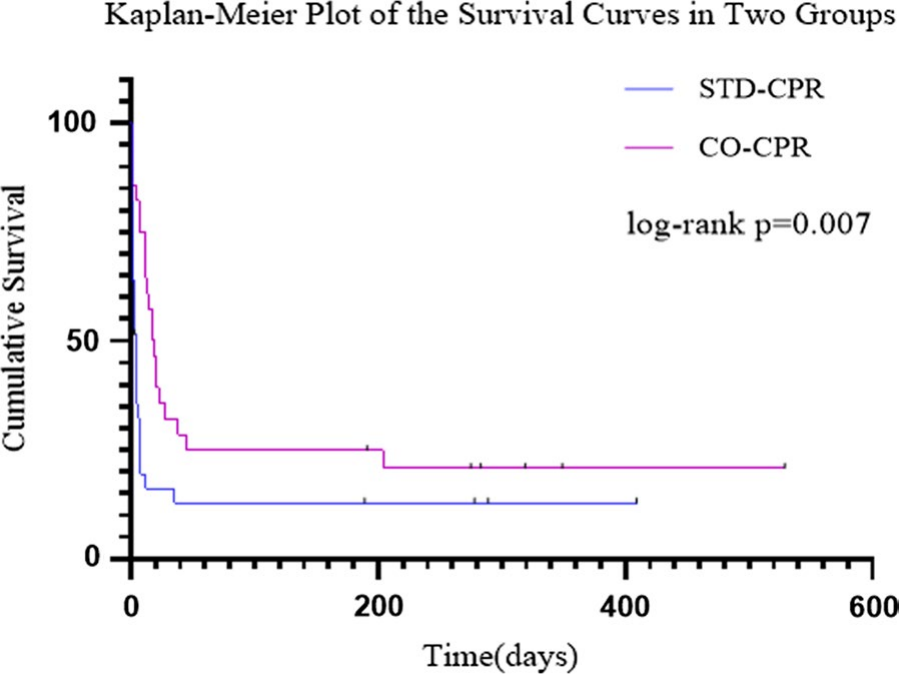
Kaplan–Meier plot of the survival curves in the CO-CPR group and the STD-CPR group at the end of follow-up

The follow-up evaluations were performed via outpatient visits for 3 patients, inpatient visits for 8 patients, and telephone calls for 23 patients. The follow-up time ranged from 1 to 529 days, and the average follow-up time was 61.7 ± 121.0 days. The Kaplan–Meier curves showed a survival benefit favouring the CO-CPR group when compared to the STD-CPR group at the end of the follow-up period (log-rank *P* = 0.007, Fig. 3).

## Discussion

In the present study, we aimed to investigate the benefit of combining conventional chest compression and abdominal compression–decompression CPR for adult OHCA patients. We found that higher proportions of patients receiving the combination method with CO-CPR had survived to hospital discharge, had better neurological outcomes at discharge according to their CPC status, and showed significant survival benefits when compared with those receiving STD-CPR.

When cardiac arrest occurs, conventional chest compression may enhance blood flow to provide sufficient oxygen to vital organs. Various methods, such as the external cardiac compressor Zoll Autopulse and LUCAS mechanical chest compression system, have been applied to improve efficiency and success rates. Unfortunately, several studies found that mechanical chest compression does not seem to improve outcomes after OHCA when compared with manual chest compression [14–16]. In addition, chest compression cannot be effectively applied under some circumstances, such as in patients with chest wall deformities, rib fractures, or haemopneumothorax.

Abdominal compression–decompression CPR was invented as an alternative compression method to augment blood return and cardiac output. Abdominal compression–decompression can change the amplitude of diaphragm motion, which plays a role in the cardiac pump, thoracic pump, and lung pump and enhances blood flow to provide sufficient oxygen to vital organs. This new method could help build circulatory and respiratory support and be used in cardiac arrest patients with sternum and thoracic rib fractures. However, little is known about whether patients with OHCA could benefit from combined chest compression with abdominal compression–decompression during CPR.

In most cases of primary cardiopulmonary arrest, blood still contains some oxygen during the early period. Conventional chest compression CPR is based on using a "thoracic pump," which is accomplished by compressing the mid-sternum, which increases the pressure inside the thorax, and blood is pumped out of the heart to the peripheral tissues, including the brain [12, 17]. In comparison, the abdominal compression–decompression technique is based on an "abdominal pump" model, which induces pressure changes within the abdominal cavity and promotes the return of blood from the abdominal cavity to fill the heart and be eventually pumped to the brain [18, 19]. A combination of abdominal compression–decompression and chest compression was previously shown to increase the venous refilling of the heart, which could generate increased coronary perfusion pressure and increase blood flow to vital organs [9, 10, 20]. With this combination method, chest release during abdominal compression leads to increased venous return to the thorax by negative intrathoracic pressure. Moreover, abdominal decompression during chest compression may lead to increased blood flow via decreased afterload. In myocardial blood flow, a better 48-h outcome was documented with the combination method compared with STD-CPR [21–24]. Hans-Richard Arntz et al. reported that the Lifestick device could perform both abdominal compression–decompression and chest compression, improve the results of CPR, and reduce the rate of injury compared to conventional resuscitation [10]. This finding suggests that patients may benefit from the combination of both chest compression and abdominal compression–decompression techniques. However, studies that compare the results and the use of different devices from different institutions might have heterogeneous results, and the results of these types of studies might have limited applicability. In our study, the combination of the two CPR methods improved the survival proportions and neurological outcomes at discharge, and there was a survival benefit at follow-up, which confirmed Hans-Richard Arntz et al.'s observations. However, the device in our study was smaller in volume, more straightforward in operation, and more accurate in terms of the operating parameters.

A significant advantage in using the new device for the combination of conventional chest compression and abdominal compression–decompression was that the instrument is small, lightweight, and easy to operate and could be suitable for hospital and nonhospital use in a variety of settings, including medical, sanitation, ambulance, rescue, and health care institutions at all levels inside and outside of the hospital.

This study has several limitations. The study was performed at a single centre. Another limitation of the study may be the relatively small number of subjects. Due to the limited sample size, most cases of cardiac arrest occurred in older patients. Autopsies were not performed in nonsurvivors; therefore, we were unable to determine whether the abdominal compression–decompression technique resulted in abdominal injuries in this study. The CO-CPR method requires an additional rescuer and STD-CPR does not.

## Conclusion

In summary, we conclude from our results that patients could benefit from the combination of conventional abdominal compression–decompression and chest compression. Clearly, a large-scale clinical trial should be performed with this promising new technique to obtain a more comprehensive evaluation of its use.

## Supplementary Information

**Additional file 1. **Sample Size Calculation in Detail.

## Data Availability

All study data are available upon reasonable request from the corresponding author and may be reused as required (We also uploaded main part of data in network of Clinical Trial Management Public Platform, http://www.medresman.org.cn).

## Abbreviations

CPR: Cardiopulmonary resuscitation
STD-CPR: Standard (or chest compression) cardiopulmonary resuscitation
CO-CPR: Combination of chest compression and abdominal compression–decompression cardiopulmonary resuscitation
OHCA: Out-of-hospital cardiac arrest
ROSC: Return of spontaneous circulation
AHA: American Heart Association
LED: Light-emitting diode
CPC: Cerebral performance category
SD: Standard deviation
COPD: Chronic obstructive pulmonary disease
