# Supplementary material for: Evaluation of abdominal compression–decompression combined with chest compression CPR performed by a new device: Is the prognosis improved after this combination CPR technique?
Source: Scand J Trauma Resusc Emerg Med. 2022 Aug 13;30:49. doi: 10.1186/s13049-022-01036-y (PMC9375386; doi:10.1186/s13049-022-01036-y)
Supplement: Supplementary file 1 — Additional file 1. Sample Size Calculation in Detail. [file 13049_2022_1036_MOESM1_ESM.docx]

In randomized design, the sample size is often calculated by,

$$n_{1}=n_{2}=\frac{\left( z_{\alpha/2}+z_{\beta} \right)^{2}\left[ p_{t}\left( 1-p_{t} \right)+p_{c}\left( 1-p_{c} \right) \right]}{\left( p_{t}-p_{c} \right)^{2}}$$

Where $p_{t}$ and $p_{c}$ are the expected proportion of return of spontaneous circulation in CO-CPR group and STD-CPR group, respectively. $Z_{\alpha/2}$ and $Z_{\beta}$ are the upper $(\alpha/2)$th and $\beta$th percentiles of the standard normal distribution, respectively. In another similar research, the minimum sample size was calculated with a $p_{t}$ value of 0.21 and a $p_{c}$ value of 0.48. In our study, we determined the expected difference in not only the proportion of return of spontaneous circulation rate, but also the neurological function and the survival between two groups in the sample size estimation. Therefore, we simplified the calculation as follows, and we usually assumed that$\alpha$ = 0.05, $U_{1-\alpha/2}=1.96$ and $d$ = 0.1.

$$n_{1}=n_{2}=\frac{u_{1-\alpha/2}^{2}\left[ p_{1}\left( 1-p_{1} \right)+p_{2}\left( 1-p_{2} \right) \right]}{d^{2}}$$

And we planned to conduct an interim analysis to re-estimate the required sample size during the trial. From our study, we briefly summarized the proportion of return of spontaneous circulation as 20% approximately in both of the groups. The sample size was re-estimated as 122 cases for each group, and the final size increased to approximately 150 cases due to a drop-out rate of 20%.
